# Structure, function, and behaviour of computational models in systems biology

**DOI:** 10.1186/1752-0509-7-43

**Published:** 2013-05-31

**Authors:** Christian Knüpfer, Clemens Beckstein, Peter Dittrich, Nicolas Le Novère

**Affiliations:** 1 Artificial Intelligence Group, University of Jena, Ernst-Abbe-Platz 2, Jena, Germany; 2 Bio Systems Analysis Group, University of Jena, Ernst-Abbe-Platz 2, Jena, Germany; 3 Le Novère lab, Babraham Institute, Babraham Research Campus, Cambridge, CB22 3AT, UK

**Keywords:** System biology, Modelling and simulation, Knowledge representation, Semantics, Meaning facets, Philosophy of science

## Abstract

**Background:**

Systems Biology develops computational models in order to understand biological phenomena. The increasing number and complexity of such “bio-models” necessitate computer support for the overall modelling task. Computer-aided modelling has to be based on a formal semantic description of bio-models. But, even if computational bio-models themselves are represented precisely in terms of mathematical expressions their full meaning is not yet formally specified and only described in natural language.

**Results:**

We present a conceptual framework – the meaning facets – which can be used to rigorously specify the semantics of bio-models. A bio-model has a dual interpretation: On the one hand it is a mathematical expression which can be used in computational simulations (intrinsic meaning). On the other hand the model is related to the biological reality (extrinsic meaning). We show that in both cases this interpretation should be performed from three perspectives: the meaning of the model’s components (structure), the meaning of the model’s intended use (function), and the meaning of the model’s dynamics (behaviour). In order to demonstrate the strengths of the meaning facets framework we apply it to two semantically related models of the cell cycle. Thereby, we make use of existing approaches for computer representation of bio-models as much as possible and sketch the missing pieces.

**Conclusions:**

The meaning facets framework provides a systematic in-depth approach to the semantics of bio-models. It can serve two important purposes: First, it specifies and structures the information which biologists have to take into account if they build, use and exchange models. Secondly, because it can be formalised, the framework is a solid foundation for any sort of computer support in bio-modelling. The proposed conceptual framework establishes a new methodology for modelling in Systems Biology and constitutes a basis for computer-aided collaborative research.

## Background

In order to understand the living nature Systems Biology develops computational models of biological systems. These models are computational in the sense, that the models are expressed in an appropriate formal language, like the Systems Biology Markup Language (SBML, [1]) and CellML [2], and can be used by computer programs in order to infer statements about its dynamical behaviour (either quantitative or qualitative). In contrast to [3] we also call differential equation models “computational”.

We call a computational model of a biological system a *bio-model* if it allows for an explanation of the mechanism behind the observed behaviour of the biological system. Therefore the model not only has to imitate the behaviour of the system. In addition, the components of the model must possess a biological meaning with respect to the modelled system. Only if the model has both the same *performance* (the behaviour) and the same *competence* (the mechanism) as the biological system, we can understand the living system by means of the model [4].

Today’s high-quality and high-throughput experimentation techniques in molecular biology are the basis for an increasing number of bio-models with growing size and complexity. Understanding biological systems on the system-level requires the integration of bio-models from different abstraction levels and with different paradigms [5]. Obviously, modelling on a system-level will require the very assistance of computers. Although computational bio-models themselves are represented in some formal language their meaning often is only described in natural language. Computer-aided modelling in Systems Biology will be impossible until the meaning of the models is formally described. In this paper we introduce the *meaning facets* of bio-models which are views of a bio-model from different perspectives. The meaning facets provide a conceptual framework for a systematic specification of the meaning of a bio-model and consequently are the basis for rigorous semantics of the bio-model.

Formal semantics of bio-models which go beyond the usual formal specification of the model structure and comprehends all meaning facets would be desirable to provide computer support in the following tasks:

### Semantics based search

Given certain desired model properties find models that exhibit these properties. For example, both example models discussed below should be retrievable by search queries of the types: “Find models describing the cell cycle!”, “Find models related to p34 protein kinase!”, or “Find models that exhibit both steady state and oscillating behaviour!”.

### Model comparison

Given two models, do they semantically overlap? Is one model a sub-model of the other? Or is one of them an abstraction of the other? In general, a method for model comparison is needed for many higher level tasks like model matching or model integration. The comparison should apply to all perspectives of the model’s meaning (see below). A comparison of two models can have different kinds of results: e.g. identical, similar, competing, contradictory, or subsuming models.

### Annotating models

The annotation of a model can be done in an interactive mode: Starting with some elementary facts about a model an interactive system (see below) infers more facts and asks for missing information. Thereby it suggests possible answers. Furthermore, the system complains about inconsistencies. The result is a complete and consistent annotation of the model.

Beside these tasks related to the storage, retrieval and exchange of models in a collaborative setting formal semantics could be the basis for computer-aided modelling. By means of automatic reasoning it would allow for higher-level tasks like:

### Model integration

Given two models that semantically overlap, what would an integrated model look like? Again, the formal semantics of the model’s components is needed in order to automate this task.

### Model use

In order to simulate and predict the behaviour of a biological system the bio-model has to be implemented in a computer code. This causes further problems: Without formal semantics a biologist must directly modify the code in order to change the model. If the extrinsic meaning of model components and their inter-dependencies with the intrinsic model structure were formalised, it would be possible to modify the model on a more abstract semantic level without the need to refer to the implementation.

### Model revision

Given desired behaviours, is the actual dynamics of a model in accordance with them? The diagnostics of a potential discrepancy will suggest possible changes of the model. The corresponding improvement could be used iteratively to “evolve” models.

A formal semantic description of bio-models would not only be useful in corresponding computer-assisted application scenarios, but also would support biologists to access models, their use and their behaviour as well as the underlying assumptions and decisions. A formal description of the involved knowledge would allow to present relevant information about a model to biologists in a familiar way.

The biological scientist does not have to cope with this rather complicated formalisation of the semantics. We envision an interactive system for computer-aided annotation of bio-models. Based on a knowledge representation system working in the background this system can guide the user in entering all the necessary information while constantly checking the consistency of the resulting information. Furthermore, the system will be able to ask for specific kinds of information depending on the information already entered and can provide candidate answers to the user.

## Results

**Figure 1:**
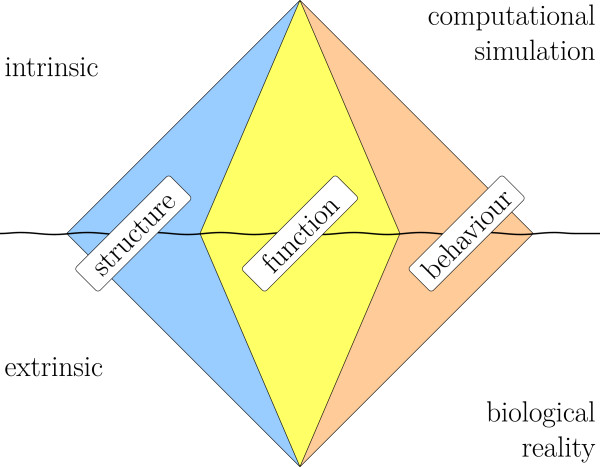
**The six meaning facets of a bio-model.** Three pairs of intrinsic/extrinsic meaning facets from left to right: structure (system, entities, relation), function (intention, instantiation, setup), and behaviour (dynamics, data, outcome). The terms in parentheses are sub-facets explained in the text.

The semantics of bio-models is a formal account of their meaning. In order to specify the semantics for the intended application scenarios we therefore have to know what a bio-model means and which aspects of its meaning are relevant. From a closer investigation of the human understanding of bio-models and the way how bio-models describe biological phenomena we derived a conceptional scheme of the meaning of bio-models [4]. This scheme resembles results from knowledge representation of complex systems (see below). The conceptional scheme consists of six meaning facets (Figure 1). The meaning facets are views at the meaning of a bio-model from different perspectives. The starting point for the interpretation of a bio-model with respect to the different facets is a model specification, i.e. an expression in some formal language. We claim that the formal semantics of a bio-model has to incorporate all of these meaning facets and the relations between them in order to enable full computer support for modelling. The proposed conceptual framework is a systematic account of the semantics of bio-models. It can guide the development of formal representations for bio-modelling and provides a coverage criterion for such efforts.

### Dual interpretation

**Figure 2:**
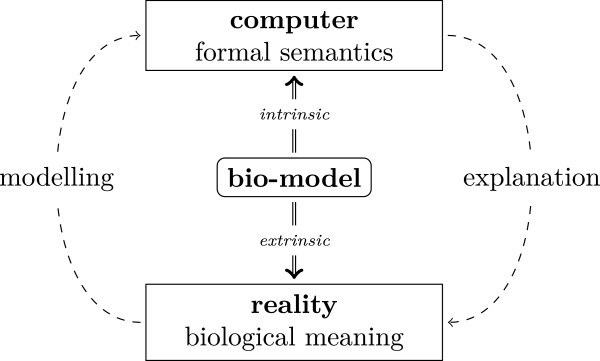
**Dual interpretation of bio-models.** A model can be mathematically interpreted as a text in a formal language resulting in “formal semantics”. This intrinsic meaning is necessary for using the model in computations. In order to exploit the results of such computations for the explanation of biological phenomena the model needs also a biological interpretation: the model possesses an extrinsic meaning relating its structure, its functionality, and its behaviour to biological reality. Ultimately, modelling is about making appropriate computational representation of biological reality.

A Bio-model has a dual interpretation: The mathematical expression bears meaning by itself without referring to the biological reality. It can be interpreted, analysed, and used in computational simulations without knowing what it represents. We call this interpretation the *intrinsic meaning* of the bio-model. However, a bio-model is more than a pure syntactical formal expression: it describes a piece of biological reality and thereby also exhibits an *extrinsic meaning*. Often, the extrinsic interpretation is referred to by the word “represents”: for example, we say that a variable *xrepresents* the concentration of a specific substance and that the oscillation shown in simulations *represents* variations in concentrations during the cell cycle. An explanatory bio-model establishes a mapping between the two conceptual sides, i.e. between the intrinsic and extrinsic meaning. Note that the biological interpretation has to be consistent with the usual conceptualisation made in biology. This ensures that modelling results represent biological phenomena in such a way that the (intrinsic interpreted) model can explain biological reality (cf. Figure 2).

In the SBML community (see, e.g., [6]) the two sides of the meaning are often called “model meaning” (all information necessary to simulate a SBML model) and “biological meaning” (annotations of what is meant by a particular SBML component). The term “model meaning”, however, is too general and therefore misleading. Furthermore, “biological meaning” is very specific to bio-models. We therefore use the terms “intrinsic” and “extrinsic” in order to (1) avoid the ambiguity of “model meaning” and (2) allow our framework to be applicable to other kinds of models.

### Three perspectives of meaning

Following research from teleological modelling in engineering (see, e.g., [7] for recent work on this topic) three pragmatic meaning perspectives can be identified: (1) The meaning regarding the components of the model and the relations between them accounts for its *structure*. (2) The meaning regarding the model in connection to its context and its intended use accounts for its *function*. (3) The meaning regarding the dynamics of the model accounts for its *behaviour*. The extrinsic/intrinsic sides of the three perspectives together form the six meaning facets illustrated in the “meaning diamond” (Figure 1). In order to represent the complete meaning of a bio-model one has to specify the intrinsic and extrinsic side of each of the three perspectives and the connections between them.

The following sections describe the three meaning perspectives in more detail. In order to illustrate the meaning facets the meaning of two semantically related models of the cell cycle from Tyson [8] is sketched with reference to existing formal approaches. The contribution of all mentioned formal approaches to the meaning facets is summarised in Table 1. Obviously, the extrinsic side is considerably less covered than the intrinsic side. This is due to the very complexity of biological reality and our restricted knowledge about it (see also the discussion of “Biological Meaning” below). The equations of the models are shown in subsection Example models, SBML encoded versions of the models can be found in BioModels Database [9]. In [10] we published a complete reconstruction of the meaning of this models which was based on a preliminary version of the meaning facets framework.

**Table 1:** Formal approaches to the meaning facets of bio-models

**Formal**	**Language element/**	**Intrinsic meaning facet**								
**approach**	**Ontology branch**	**Structure**			**Function**			**Behaviour**		
		**(S1)**	**(S2)**	**(S3)**	**(F1)**	**(F2)**	**(F3)**	**(B1)**	**(B2)**	**(B3)**
SBML	species		x							
	reaction			x						
	kineticLaw			x						
	initialAmount					x				
	parameter					x				
SBO	“modelling framework”	x								
SED-ML	Simulation				x					
	Change					x				
KiSAO	“modeling and simulation						x			
	algorithm”									
DYML	feature							x		
	constraints					x				
TEDDY	“temporal behaviour”							x		
	“behaviour diversification”							x		
Temporal logics								x		
SBRML	Result								x	x
Fielded Text									x	x
**Formal**	**Language element/**	**Extrinsic meaning facet**								
**approach**	**Ontology branch**	**Structure**			**Function**			**Behaviour**		
		**(S1)**	**(S2)**	**(S3)**	**(F1)**	**(F2)**	**(F3)**	**(B1)**	**(B2)**	**(B3)**
UniProt			x							
NCBI Taxonomy		x								
Database										
Gene ontology	“Biological Process”	x						x		
SBO	“physical entity representation”		x							
	“systems description parameter”		x							
	“occurring entity representation”			x						
	“systems biology representation”			x						
Reactome				x						
Cell type						x				
Ontology										
SABIO-RK						x				
FuGE	Material					x				
	Investigation						x			
	Data								x	x
SBRML	Result								x	x
Fielded Text									x	x

Formal approaches (languages, ontologies and biological resources) for computer representations of bio-models and their contribution to the formalisation of the meaning facets as shown for the example models by Tyson. An “x” means that the respective formal approach or some given aspect (language element or ontology branch) of it is able to represent the specific meaning facet or at least parts of it. The table is divided into intrinsic (top) and extrinsic (bottom) meaning facets.

### Example models

In this section we introduce two models of the cell cycle by Tyson [8] which are used as an example in the following description of the meaning facets. Both models describe the formation and activation of the maturation promoting factor (MPF), a hetero dimer made of the two proteins cyclin and cdc2.

*Model 1* consists of six ordinary differential equations (ODEs) where each equation models the temporal evolution of the concentrations of one of the involved substances with respect to the concentrations of the other substances:

(1) $$\begin{array}{ll} \;d[\;\text{C2}]/dt & =k_{6}[\;\text{M}]-k_{8}[∼\text{P}][\;\text{C2}]+k_{9}[\;\text{CP}]\; \end{array}$$

(2) $$\begin{array}{ll} \;d[\;\text{CP}]/dt & =-k_{3}[\;\text{CP}][\;\text{Y}]+k_{8}[\;∼\text{P}][\;\text{C2}]-k_{9}[\;\text{CP}]\; \end{array}$$

(3) $$\begin{array}{ll} \;d[\;\text{pM}]/dt & =k_{3}[\;\text{CP}][\;\text{Y}]-[\;\text{pM}]F([\;\text{M}])+k_{5}[\;∼\text{P}][\;\text{M}]\; \end{array}$$

(4) $$\begin{array}{ll} \;d[\;\text{M}]/dt & =\;[\;\text{pM}]F([\;\text{M}])-k_{5}[\;∼\text{P}\;][\;\text{M}]-k_{6}[\;\text{M}]\; \end{array}$$

(5) $$\begin{array}{ll} \;d[\;\text{Y}]/dt & =k_{1}[\;\text{aa}]-k_{2}[\;\text{Y}]-k_{3}[\;\text{CP}][\;\text{Y}]\; \end{array}$$

(6) $$\begin{array}{ll} \;d[\;\text{YP}]/dt & =k_{6}[\;\text{M}]-k_{7}[\;\text{YP}]\; \end{array}$$

(7) $$\begin{array}{ll} F([\;\text{M}]) & =k_{4}^{\prime }+k_{4}{\left([\;\text{M}]/[\;\text{CT}]\right)}^{2}\; \end{array}$$

Involved substances are: cdc2 (C2), phosphorylated cdc2 (CP), inactive MPF (pM), active MPF (M), cyclin (Y), phosphorylated cyclin (YP), adenosine triphosphate (∼P), and amino acids (aa). CT means total cdc2, i.e. [ CT]=[ C2]+[ CP]+[ pM]+[ M]. The *k*_*i*_ are kinetic rate coefficients.

*Model 2* is a mathematical abstraction of *Model 1* under certain additional biological assumptions:

(8) $$\begin{array}{ll} du/dt & =k_{4}(v-u)(\alpha +u^{2})-k_{6}u\; \end{array}$$

(9) $$\begin{array}{ll} dv/dt & =(k_{1}[\text{aa}]/[\text{CT}])-k_{6}u\; \end{array}$$

(10) $$\begin{array}{ll} u & =\;\;[\;\text{M}]/[\;\text{CT}\;]\; \end{array}$$

(11) $$\begin{array}{ll} v & =([\;\text{Y}]+[\;\text{pM}]+[\;\text{M}])/[\;\text{CT}]\; \end{array}$$

(12) $$\begin{array}{ll} \alpha & =k_{4}^{\prime }/k_{4}\; \end{array}$$

*u* and *v* are relative concentrations following the given equations.

### Structural facets

A bio-model describes state changes of a formal system. The notion of structure refers to the aspects of the system which do not change. In most general terms structure can be described by entities having attributes and relations between the entities: the attributes of the entities constitute the state of the system, the relations describe inter-dependencies between the attributes of related entities. The structural entities and relations have to be rather classes than individuals. Whereas a individual molecule can be formed, changed and destroyed, a molecule sort (a class of molecules) remains the same all over the time. Based on the relations a programme determines how the system state is changing. The intrinsic structural meaning is obtained by interpreting the given model specification with respect to the formalism used. An explanation of the behaviour of the modelled biological system requires to map this intrinsic structure to relevant biological objects characterised by quantities and interactions establishing mechanisms. Usually, what is called a “biological systems” in fact denotes a class of concrete systems in reality. The concrete systems are considered on a specific conceptual level, e.g. as gene regulatory networks, protein interaction networks, signal transduction pathways, or metabolic networks. In turn, this common view onto the concrete systems establishes itself an abstract system, the “biological system”. The conceptual level must be reflected by the formalism used. In detail the structural meaning can be characterised as follows:

#### (S1) System

##### intrinsic

Which *formal system* is specified by the encoded model (the model itself)? Which *formalism* is employed by the model (modelling framework, spatiality, stochasticity)?

##### extrinsic

Which *biological system* corresponds to the formal system (species, cell type, biochemical system)? Which *conceptual level* is reflected by the used formalism (system type, granularity, spatial and temporal resolution)?

#### (S2) Entities

##### intrinsic

What are the *entities* of the formal system (individuals, collections, agents)? Which *attributes* of the entities describe the state of the system (variables, terms)?

##### extrinsic

What biological *objects* correspond to the model entities (molecules, substances, cells)? Which *quantities* (amounts, concentrations, units) correspond to the model attributes?

#### (S3) Relations

##### intrinsic

What are the *relations* between the entities (inter-dependencies, correlation, neighbourhood)? What is the *programme* describing changes of the attributes of related entities (operations, equations, update rules)?

##### extrinsic

What biological *interactions* correspond to the model relations (reactions, transformations, diffusion)? What biological *mechanisms* realising the interactions between objects correspond to the model programme (reaction steps, bonding, activity)?

#### Notes

The specification of the formalism (S1) will restrict the ways one can use the model. This information is essential for the interpretation of a model specification as a formal system. If, for example, a model specification does not provide information about the intended modelling framework (like discrete and continuous) the specification of the formal system is incomplete (see [11]). However, with this information it will also be possible to automatically convert models from one modelling frameworks in another [11]. Fages and Soliman [12] investigate the different interpretations of SBML models depending on the chosen modelling framework and relate the resulting different semantics by the theory of abstract interpretations.

Biological systems are hierarchically organised. Often, this is reflected by a partonomy of entities in a model. This partonomy is described as relations between the entities (S3). Furthermore, it has to be described how attribute changes in a part influence attribute changes in the corresponding whole and vice versa. Thereby the whole system can be seen as the top-level entity in the partonomic hierarchy.

In general, the programme has formal parameters. The actual parameters (i.e. the parameter values) must be appropriately instantiated, see facet (F2) below.

For understanding a model it is useful to capture the relationships between biological objects, e.g. between a protein and its phosphorylated versions or between a dimer and its part (maybe modelled as partonomic relations, see above).

Biological processes often happen in separated compartments. There are two ways to account for this compartmentalisation: In which compartment an object resides can be represented by an attribute of the corresponding entity. In contrast, objects of the same type residing in different compartments can be modelled by different classes of entities. A relation has to describe the exchange between the compartments.

#### Example

Intrinsically, both Tyson models are encoded in SBML. The respective *formal systems* (S1) are given by the equations in subsection Example models. The used *formalism* (S1) can be characterised as a set of coupled ordinary differential equations of continuous state variables in the common independent variable *t*, which describes a deterministic non-spatial state evolution. The modelling framework can be specified by a term from the Systems Biology Ontology (SBO, [13]): “non-spatial continuous framework” (SBO:0000293). The intrinsic structural meaning of SBML models is formalised by the SBML specification (we use typewriter font for SBML keywords): The *entities* (S2) are given as species in the listOfSpecies. Each species has an unique id and a name, e.g. the species C2 is called “cdc2k”. Each id is also used as dependent variable within the kineticLaws (see below), representing the amount as an *attribute* (S2) of the according species. The *relations* (S3) are reactions in the listOfReactions. Each reaction has a kineticLaw describing the corresponding changes of the species amounts. There can be (formal) parameters in the kineticLaw. For instance there is the following reaction (Reaction1 in the SBML encoding) in the Tyson model:

$$M\overset{k_{6}}{\underset{}{\to }}C2+\text{YP}$$

with the kinetic law *k*_6_[ M], where *k*_6_ is a parameter determining the reaction rate. The *programme* (S3) of an SBML model is just the set of ODEs (cf. the equations in subsection Example models) reflecting the kinetic laws of the single reactions. For better legibility, we use the common style for kinetic equations with square brackets denoting the amount of a species. However, SBML has a specific syntax for kineticLaw based on MathML [14].

Extrinsically, both Tyson models describe a *biological system* (S1) of MPF (maturation promoting factor) formation and activation which controls major events of the cell cycle in different organism: frog, sea urchin, and fission yeast. The extrinsic meaning of each SBML tag can be given by annotations pointing to an appropriate description of biological knowledge. E.g. C2 represents the biological *object* (S2) “Cyclin-dependent kinase 1” for which the UniProt [15] entry P04551 can be given. MIRIAM Registry [16] can be used for a unified way of referring to all external resources used in describing the meaning of bio-models. For example, the UniProt entry for the extrinsic meaning of C2 will become urn:miriam:uniprot:P04551. By means of identifier.org [16] one can also provide a persistent URL for this information: http://identifiers.org/uniprot/P04551z. The addressed organism could be assigned by the NCBI Taxonomy Database [17], e.g. sea-urchins have the Taxonomy ID: 7625. But one could also use more general entries, like the common parent term of sea urchins and frogs Deuterostomia (Taxonomy ID: 33511), or even more general Eumetazoa (Taxonomy ID: 6072). It would not be suitable to go further up in the taxonomy, because the facts about embryonic development in [8] do not apply in general for higher taxa. An annotation can specify the addressed biochemical system. The addressed system of the example models can be specified by a link to the “mitotic cell cycle” (GO:0000278) entry of the Gene Ontology (GO, [18]). The biological systems are regarded on the *conceptual level* (S1) of pools of molecular entities without consideration of spatial effects. The justification for this are high enough numbers of molecules and fast diffusion. *Model 1* is a network of protein-protein interactions, where catalytic reactions between proteins changes their concentrations in time (extrinsic interpretation of the independent variable *t*). *Model 2* is an abstraction of the actual protein-protein interactions. In addition to the SBML annotations, it is possible to refer to SBO directly within a SBML tag. SBO terms can be used for a top-level classification of molecules and reactions. For example, C2 can be classified as “polypeptide chain” (SBO:0000252), and Reaction1 as “dissociation” (SBO:0000180). Each involved substance has as a *quantity* (S2) its molar concentration (SBO:0000472) in mol/1. Reaction1 represents the *interaction* (S3) “cyclin cdc2k dissociation” for which the Reactome entry REACT_6308 can be given. Furthermore, the role which a molecule plays in a reaction can also be described by SBO (e.g. reactant, product, modifier). The *mechanism* (S3) underlying most of the reactions is mass-action kinetics, which could be annotated by “mass action rate law” (SBO:0000012). Only Reaction9 represents a special mechanism for a autocatalytic feedback [8].

The structural meaning of *Model 2* can be expressed in a similar way. However, the extrinsic meaning of u and v is not straightforward. It can only be derived using the defining equations *u*=[ M]/[ CT], *v*=([ Y]+[ pM]+[ M])/[ CT] and assigning meanings to the contained entities like M (see above). The extrinsic interpretation of the reactions of *Model 2* is even harder. As interpreted in BioModels Database the reactions do not contain a feedback loop anymore but still showing oscillating behaviour!

### Functional facets

A bio-model is simulated in order to get data for answering biological questions. The function of a model describes how the model structure is intended to be used in simulations to generate dynamic behaviour. Before a model can be used in simulations it has to be fully instantiated, i.e. all parameters should be given actual values and the initial state of the model has to be set. The simulation setup describes the exact procedure applied to the model instance. In addition, the post-processing describes how to produce the final outcome. The instantiation and the setup of the simulations performed with the model have to reflect the specific boundary conditions and the experimental settings under which the biological system is observed. The functional meaning can be characterised by the following questions:

#### (F1) Intention

##### intrinsic

What is the *intended use* of the model (simulation type, combination of simulations, desired outcome)? Which *constraints* are imposed on the model (value restrictions, ratios, conservation rules)?

##### extrinsic

Which biological *questions* are addressed to the model (explanation, hypothesis testing, exploration, dependency analysis)? Which *assumptions* provide the basis for the constraints (likelihoods, justification, evidence)?

#### (F2) Instantiation

##### intrinsic

Which *instantiation* of the model is used for the simulation (parameter values, parameter ranges)? Which *initial values* are chosen for the entities attributes (value assignment to variables)?

##### extrinsic

Which *boundary conditions* correspond to the model instantiation (environment, kinetic data, plausible ranges)? Which *initial state* of the biological system corresponds to the initial values set for the model (initial concentrations)?

#### (F3) Setup

##### intrinsic

Which *setup* is used for simulation experiments (simulation algorithm, algorithm settings, perturbations)? Which *post-processing* of the raw simulation data generates the desired outcome (normalisation, conversations of units, calculations)?

##### extrinsic

Which biological *experimental settings* correspond to the setup for simulations of the model (experimental protocol)? Which *result calculation* produces the requested results of the experiment (normalisation, conversations of units, calculations)?

#### Example

In the following we describe the functional aspects of using *Model 1* for producing the time series of Figure 3(a) in [8].

**Figure 3:**
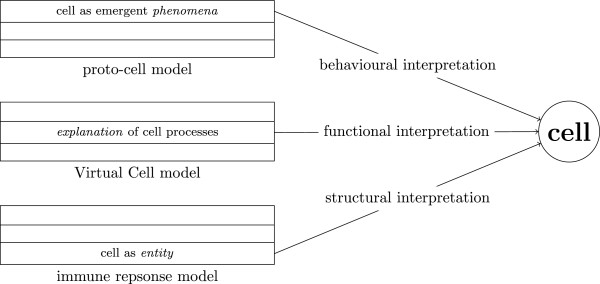
**Different meanings of “Cell”.** Three different examples of models for which the extrinsic meaning “Cell” can be viewed at from different perspectives: a proto-cell model [30], a Virtual Cell model [31], and an immune response model [32].

Intrinsically, the functional meaning of the example models is equivalent to a complete description of simulation experiments applied to the model comprising all the details of (F1-3). Most simulation tools use their own proprietary format to encode this information which hampers the reuse of functional information. In order to overcome this situation the Simulation Experiment Description Markup Language (SED-ML) is developed [19]. In a SED-ML description algorithm used for the simulation can be specified using KiSAO (Kinetic Simulation Algorithm Ontology, [13]). The *intended use* (F1) is the generation of a time series through numerical integration of the model. Thereby the time evolution of the amounts of M and YT are reported. In SED-ML this intended type of simulation can be set in the listOfSimulations as uniformTimeCourse. Tyson gives some *constraints* (F1), e.g. [ CT]=*const*. and *k*_2_≪*k*_3_[ CT]. Constraints often are only implicit in the chosen simulation paradigm and algorithm; making them explicit will be a future challenge. It is an open issue how to formalise such constraints and the corresponding assumptions (see below). A combination of Constraint-Logic-Programming [20] with languages from Systems Biology seems to be a promising research direction for explicitly incorporating constraints and assumptions into models. For the *instantiation* (F2) of the model Tyson gives parameter values. Parameters can be set in SBML via the corresponding value attribute. There are no *initial values* (F2) explicitly given in [8]. However, one can find appropriate initial values from the time series in Figure 3(a). The SBML file from BioModels Database contains such an assignment in the initialAmount attributes. The parameters and initial values can also be set or modified in SED-ML. In SED-ML it is possible to describe the *setup* (F3) of the experiment and the *post-processing* (F3) of the data: For Figure 3(a) of [8] a uniform time course from time 0 min to 100 min with a step size of 0.001 min is produced with a fourth-order Adams-Moulton integration routine (KISAO_0000280). Subsequently, the raw amount data of M and the sum of all “cyclin” entities (called “total cyclin”, [ YT]) are normalised by dividing by the amount of CT.

Tyson explicitly states biological *questions* (F1) addressed to the model. The corresponding question for Figure 3(a) is: “Can the same model also account […], for rapid cycles of DNA synthesis and cell division (without cell growth) during the embryonic cell cycle, […]” [8], p.7329. That is, Tyson tries to explain a specific biological phenomenon. At the moment there is no way to formalise this question. However, we could imagine a classification of modelling aims. For each modelling aim appropriate simulation types could be identified. Tyson indicates *assumptions* (F1) as the basis for the parameter constraints. For example, he assumes that cdc2 is constantly synthesised in growing cells which supports [ *CT*]=*const*. The concrete *boundary conditions* (F2) corresponding to the instantiated model for Figure 3(a) are the conditions found in early embryonic cells [8]. The Cell Type Ontology [21] can be used to specify the cell type: “early embryonic cell” has the ID CL:0000007. Tyson states that there is no experimental kinetic data available for this situation. Also, there is no *initial state* (F2) of the biological system given by Tyson. If such data would exist the corresponding SBML parameter and initialAmount could be annotated with kinetic data entries from appropriate sources (e.g. SABIO-RK, [22]). As with kinetic data and the initial state of the biological system, Tyson does not provide any information about *experimental settings* (F3) and the following *result calculation* (F3). Nevertheless, there are some standards for describing experimental protocols, like FuGE [23] for functional genomics experiments.

The functional facets of *Model 2* can be described in a similar way. However, there are specific assumptions underlying the abstraction of *Model 2* from *Model 1*. This assumptions are mainly reflected by the structure of *Model 2* (S2,3) and have to be met in a corresponding use of *Model 2* (F1,2).

### Behavioural facets

A bio-model is used in simulation experiments in order to investigate its behaviour. A simulation experiment produces raw numerical data. Often, this data is post-processed into the final desired outcome of the experiment. The model dynamics is a qualitative description of this experimental outcome. From the behavioural perspective the model dynamics should correspond to observed biological phenomena. The observed biological phenomena are supported by experimental results which are obtained from measurements of the biological system in question. The behavioural meaning can be characterised by the following questions:

#### (B1) Dynamics

##### intrinsic

Which types of *dynamics* does the model show in simulations (fixed points, periodic behaviours, chaotic behaviour)? Which *diversification* in the dynamics does the model possess (stability, bifurcations)?

##### extrinsic

Which biological *phenomena* correspond to the model dynamics (cyclic behaviour, steady state)? Which *variability* of the biological phenomena correspond to the diversification in the dynamics of the model (switching behaviour, excitability)?

#### (B2) Data

##### intrinsic

Which *raw data* does the model produce in simulations (series of values)? Which *index* is used for the raw data (modelling time, parameter value, initial value)?

##### extrinsic

Which experimental *measurements* correspond to the yielded raw data (series of values)? Which *key* is used to identify the single measurements (time, conditions, initial states)?

#### (B3) Outcome

##### intrinsic

What is the *outcome* of the simulation (specific values, time courses, phase portraits, bifurcation diagram)? Which *characteristics* of the model dynamics can be identified (maximal and minimal values, periods, Lyapunov exponents)?

##### extrinsic

Which experimental *results* correspond to the outcome of the simulation (specific values, time courses, phase portraits, bifurcation diagram). Which *observables* correspond to the characteristics of the models dynamics (maximal and minimal concentrations, cycle length, stability)?

#### Example

For both example models Tyson identified three different types of *dynamics* (B1) dependent on the parameters setting: stable steady state, spontaneous limit cycle oscillation and excitable switch [8]. The intrinsic meaning of this dynamics types can be formalised by terms from the Terminology for the Description of Dynamics (TEDDY, [13]), e.g. TEDDY_0000113 “Stable Fixed Point” for the stable steady state. TEDDY also provides terms for *diversification* (B1) of dynamics: In the example model there is a supercritical Hopf bifurcation (TEDDY_0000074) between the steady state and the oscillation if parameters *k*_4_ and *k*_6_ are varied. TEDDY only provides the vocabulary for describing the dynamics of models. We also need a language for relating conditions and types of dynamics. In an envisioned Dynamic Markup Language (maybe called DYML) the dynamics of *Model 2* could be formalised as follows (simplified notation): 

Note that only the parameters *k*_4_ and *k*_6_ are allowed to vary. All other parameters are set as in [8], Table two, p.7329. The constraints are taken from the phase plane analysis in [8], p.7332.

There exist other approaches for qualitative descriptions of model behaviour like temporal logics [24]. In BIOCHAM [25] a temporal logic is used as a query language for properties of the dynamics of bio-models. Instead of numerical simulations model checking techniques (see, e.g., [24]) can be used to answer such queries. In [26] a temporal logic extended by constraints over real numbers is used to express quantitative properties of temporal behaviour and to optimise parameters. Such quantitative temporal logics are worth to be considered as possible candidates for the needed model dynamics language.

The identification of the dynamics of the example models is based on simulation experiments which produce as *raw data* (B2) series of amount values for each entity. The *index* (B2) for these values is the modelling time (*t*). The intrinsic meaning of the values are the values itself. However, there is an issue to relate – in a formalised manner – the single values of a result table to the model attributes and the corresponding condition. There are some approaches to establish this connection, like SBRML [27] and Fielded Text [28]. The *outcome* (B3) of the simulations are plots of [ M]/[ CT] and [ YT]/[ CT] (see (F3) in the example above) against modelling time under different settings. Tyson also reports some *characteristics* (B3) of the model dynamics, e.g. relative amounts of M in steady state and period of the oscillation. Beside the time series there is another outcome in [8]: in the space of the parameters *k*_4_ and *k*_6_ regions of different qualitative behaviour are identified. Each region represents classes of concrete times series with common properties. These classes are the different dynamics of the model mentioned above and the plot of the regions in parameter space visualises the diversification in the dynamics.

Concerning experimental *measurements* (B2) there is the same issue of connecting measurement values and the corresponding *keys* (B2) with model attributes and conditions as for simulation results (see above). Tyson does not provide concrete experimental measurements or *results* (B3). Instead, he refers to conclusions drawn from such data. For instance, he characterises the phenomenological *variability* (B1) by the different modes of operation observed in different developmental stages and states typical *observables* (B3) of these modes like the period of division cycles. The three different *phenomena* (B1) are mapped to the types of dynamics of the model: metaphase arrest in unfertilised eggs is represented by the steady state, rapid division cycles in early embryos by the spontaneous oscillation, and the growth-controlled division cycles in non-embryonic cells by the excitable switch. The extrinsic meaning of the dynamics can be grounded in external resources, e.g. “cell cycle arrest” (GO:0007050) for the metaphase arrest. The variability can be represented by linking conditions (e.g. early embryo stage) with the specific phenomena observed under this conditions.

The behaviour of *Model 2* is the same as for *Model 1* except for the number of dimensions of the dynamical system. Indeed, the simplified *Model 2* is used in [8] in order to also analyse the dynamics of *Model 1*.

### Global meta-information

Beside the meaning of a model itself there exists additional information describing the role of the model in scientific research. We call meta-information of this type “global meta-information”. Global meta-information accounts for the origin of the model, the access to the encoded model in some formal language, and the relation of the model to other models. We will not provide a detailed systematics of global meta-information here. Instead we describe just the global meta-information for the example models.

#### Example

Both example models are originally published in [8]. The corresponding meta-information for the *origin* of the models comprises the paper itself (PubMed ID: 1831270), its author (John J. Tyson) and its date of publication (August 1991).

Important meta-information for the *access* of an encoded model involves the place (file name, URL, database ID), the used format (e.g. SBML, CellML), and the date and author of the encoding. If the model is stored in a database then there also exists meta-information about the curation process (curators, date, last modification). The example models are available in BioModels Database encoded in different formats: *Model 1*http://identifiers.org/biomodels.db/BIOMD0000000005*Model 2*http://identifiers.org/biomodels.db/BIOMD0000000006

BioModels Database also lists the mentioned meta-information about the encoding and curation process. For example, one can access the encoded models in SBML, Level 2, Version 4. The format is determined by the xmlns:m attribute in the sbml tag.

A model can have different *relations* to other models: It can be evolved from preliminary versions, it can be abstracted or integrated from other models, and it can be compared to competing models. The derivation of *Model 2* from *Model 1* is the result of an abstraction relation between the two models. Tyson also mentioned some existing related model. For example, he states that *Model 2* is a modified version of the famous “Brusselator”. There are formal approaches to relate models, e.g. based on graph theory [29].

## Discussion

Our analysis showed that formalising the meaning of bio-models requires a significant effort and is not trivial, since the meaning appears from several perspectives and in different facets (cf. Figure 1). We have nevertheless demonstrated how, in principle, it is possible to specify the meaning in a form that is understandable by both, computers and humans.

The proposed meaning facets framework allows for a systematic classification of existing approaches for computer-readable representations of model meaning. The framework therefore can be used to evaluate the coverage of representations and to identify missing pieces. Interesting next steps involve the extension of *BioModels Database*[9] by introducing the behavioural meaning perspective and by considering the intrinsic mathematical structure in order to grasp the semantics of variables like *v* in *Model 2*.

For the envisioned intelligent computer-aided working environment, which semantically guides model design and use and fosters the development of sound and well annotated bio-models, we have to establish appropriate languages for the missing pieces, like a description language for the behavioural perspective. Furthermore, existing languages and resources have to be improved in order to enable the necessary reasoning capabilities. The proposed meaning facets framework can direct this developments.

### Biological meaning

The following are some explanatory notes regarding the biological (extrinsic) meaning of bio-models and its formalisation: 

1. In general, the extrinsic meaning will only be partial, i.e. there may be aspects of the model without counterparts in the biological world. But at least there has to be some aspect of a model which has an extrinsic interpretation. Without representing a concrete biological system a model would be (biological) meaningless!

2. Even if an extrinsic interpretation of some model aspect exists it doesn’t have to be intuitive. The more intuitive a model represents our perception of reality, the better it explains the modelled system and consequently contributes to an understanding of the living nature.

3. The extrinsic interpretation depends on the intention of the model. Therefore the same mathematical construct can have more than one biological meaning. For example, the exponential grow $\dot{x}=\mathrm{\alpha x},\alpha >0$ can be a model for different biological phenomena.

4. It is tempting to assume that familiar biological objects, like “cell” are represented in the model from the structural perspective, i.e. that there is a structural entity interpreted as “cell”. This often is not the case. Figure 3 illustrates that all three perspectives of meaning can refer to “cell”. This shows, that biological objects can also have some behavioural and functional aspects. The three meaning perspectives should not be regarded as independent from each other, but rather as different views of an indivisible unity.

### Related work

The insight in the dual interpretation of mathematical models are of course not new: The “knowledge representation hypothesis” [33] demands that any useful formal representation needs both: to play a formal role and to have an “external semantical attribution”. Also, Simon’s notion of artefacts (like models) as interfaces between an inner and an outer environment [34] resembles the dual interpretation of bio-models. However, for a systematic formal specification of the meaning of bio-models it is very useful to distinguish between the intrinsic and the extrinsic interpretation. In fact, Rosen’s central “Modeling Relation” [35] is formulated as a congruence between a natural system and a formal system (a model). Thereby, biological “percepts” and “linkages” between them are encoded by formal entities and relations. Inferences in the formal systems can be decoded as predictions about the behaviour of the natural system. Thus, [35] already distinguish between the intrinsic/extrinsic sides on the structural and the behavioural perspective with a focus on the interplay of the two sides, not on the details of the structure and behaviour provided in this paper.

There is a similar distinction between perspectives in [36]: Their “model description” is more or less what we call structural perspective. Their “simulation description” is part of the functional perspective described above. Our behavioural perspective is called “simulation results description” in [36]. Our meaning facets however are more systematic and provide more details from each perspective. [36], nevertheless, gives a good overview of important standards, languages, and ontologies for the three perspectives.

Another systematic approach to models is Zeigler’s “framework for modeling and simulation” [37]. The framework consists of four elements: the source system, the experimental frame, the model, and the simulator. Each element involves knowledge on specific “system specification levels” (for details cf. [37]). There are some connections between Zeigler’s framework and the meaning facets: Zeigler’s “state transition” level 3 [37], p.17f corresponds to the *programme* in (S3), the “coupled component” level 4 corresponds to *entities* (S2) and *relations* (S3). Both levels together are used to specify models, therefore a “model” in Zeigler’s framework is what we call *structural facets*. The “experimental frame” formalises the conditions for simulating the model, thus it corresponds to the *instantiation* (F2) and the *setup* (F3). Zeigler claims that the experimental frame “is a operational formulation of the objectives that motivate a modeling and simulation project” [37], p.27, so it corresponds also to the *intention* (F1). The “source system” is regarded as a source of data of the “I/O behaviour” level 1, which corresponds to *raw data* (B2). Although there are parallels between the two frameworks, Zeigler’s work is focused on the mathematical side of building models and using them in simulations. In contrast, the approach proposed here regards models as “integrators of knowledge” [13] in the centre between computations and biological reality (cf. Figure 2). As a consequence our conceptual framework provides a detailed account of the extrinsic meaning from different perspectives on models. Klir [38] also classifies the knowledge about investigated systems. He establishes what he calls “epistemological levels of systems” which are very similar to Zeigler’s system specification levels. In fact, Zeigler starts his presentation with a review of Klir’s levels and shows the correspondence with his approach [37], p.11ff.

The SemSim (for “semantic simulation”) project [39] aims to support integration of bio-models by means of their semantics. In SemSim models are annotated from the structural perspective with links to different biological ontologies [40]. Additional, they use the Ontology of Physics for Biology (OPB, [41]) to describe the physical quantity represented by a model variable.

In [42] there is a distinction between function as mediating between structure and behaviour and function as purpose. The first determines the “structural behaviours”, i.e. all possible behaviours the model is able to show. The second restricts the possible behaviours to the “expected behaviours” which are intended by the modeller making function “the bridge between human intention and physical behavior of artifacts” [43], p.271. The distinction between structural and expected behaviours originates from [44]. In this paper function is seen as purpose. Thus, the behaviour perspective describes expected behaviours.

## Conclusion

In this paper, we present a systematic in-depth account of the semantics of bio-models. We show, that the meaning of bio-models has intrinsic and extrinsic aspects which can be viewed at from three perspectives: the structure, the function, and the behaviour of the model. The resulting six meaning facets provide a conceptual framework for the formalisation of the knowledge involved in building and using bio-models.

The proposed conceptual framework is a suitable foundation for computer-aided annotation, integration, and retrieval of bio-models. Obviously, this is only a first step in solving the “semantic puzzle” of formalising the meaning of bio-models. The framework helps in identifying how do the missing pieces look like and how they are fit together.

Our meaning facets are also a way for structuring and clarifying our understanding of bio-models. They can guide the model builder during the model building process and can assist the model user in comprehending models. In fact, the meaning facets framework establishes a new methodology for computer-aided collaborative modelling in Systems Biology.

## Competing interests

The authors declare that they have no competing interests.

## Authors’ contributions

CK originally developed the conceptual framework in continual discussion with the other authors. CB helped to theoretically analyse the knowledge involved in modelling and how to formalise it. NLN brought in the demand and relevance of semantics for bio-models. PD helped in structuring the meaning facets and with the case study. All authors read and approved the final manuscript.
